# Radiation Plus Anti-PD-1 Therapy for NSCLC Brain Metastases: A Retrospective Study

**DOI:** 10.3389/fonc.2021.742971

**Published:** 2021-10-21

**Authors:** Guixiang Liao, Yuting Qian, Sumbal Arooj, Zhihong Zhao, Maosheng Yan, Zihuang Li, Hongli Yang, Tao Zheng, Gang Li, Xianming Li, Muhammad Khan

**Affiliations:** ^1^ Department of Oncology, Shenzhen People’s Hospital, The First Affiliated Hospital Of Southern University Of Science And Technology, Shenzhen, China; ^2^ Department of Radiation Oncology, Shenzhen People's Hospital, The Second College of Jinan University, Shenzhen, China; ^3^ Department of Biochemistry and Molecular Biology, University of Sialkot, Sialkot, Pakistan; ^4^ Department of Nephrology, Shenzhen People’s Hospital, Second Clinical Medicine Centre, Jinan University, Shenzhen, China; ^5^ Department of Chemoradiation Oncology, The First Affiliated Hospital Of Wenzhou Medical University, Wenzhou, China; ^6^ Department of Oncology, The First Affiliated Hospital of Anhui Medical University, Hefei, China

**Keywords:** non-small cell lung cancer (NSCLC), immunotherapy (IT), anti-PD-1 immunotherapy, whole brain radiation therapy (WBRT), immune checkpoint blockade (ICB), brain metastasis (BM)

## Abstract

**Background:**

Radiation therapy (RT) is the mainstay of brain metastases (BMs), and anti-PD-1 blockade has led to intracranial responses in non-small cell lung carcinoma (NSCLC) patients with BMs.

**Objective:**

This study aimed to evaluate the efficacy and safety of adding anti-PD-1 blockade to RT in the management of NSCLC patients with BM in terms of survival outcome.

**Materials and Methods:**

We retrospectively reviewed 70 NSCLC patients with BMs who were treated with whole brain radiation therapy (WBRT) between January 2016 and January 2021. Of the 70 patients, 29 additionally received anti-PD-1 therapy within 30 days of WBRT initiation. Baseline characteristics of the patients and efficacy outcomes such as progression-free survival (PFS) and overall survival (OS) were statistically compared using SPSS v26. Results were obtained using the Chi-square test/Fisher exact test, t-test, Kaplan-Meier, and Cox regression survival analyses.

**Results:**

The median survival for the entire cohort was 24 months (95% CI, 19.5–28.5). The median survival times for WBRT alone and WBRT plus anti-PD-1 therapy cohorts were 20 months (95% CI, 11.6–28.3) and 27 months (95% CI, 19.5–28.5), respectively (*p=0.035*). There was no statistical difference in PFS for the treatment cohorts (median PFS for WBRT alone: 7 months *vs*. 12 months for WBRT plus anti-PD-1, *p=0.247*). In EGFR wild-type subgroup (n=31), both PFS (*p=0.037*) and OS (*p=0.012*) were significantly improved. Only the treatment group (WBRT plus anti-PD-1) was a significant predictor of OS on univariate and multivariate analyses (*p=0.040*). There were no significant differences in adverse events among the treatment groups.

**Conclusions:**

NSCLC patients with BM receiving additional anti-PD-1 therapy may derive better OS than WBRT alone without any increase in adverse events. Prospective well-designed studies are warranted to validate and elucidate the additive effects of the two modalities in this group of patients.

## Introduction

Lung cancer is the second most common cancer type in terms of incidence rate (T: 228820, 12.7%; M: 116, 300 13%; F: 112, 520 12%), and is the leading cause of death (T: 135720, 22%; M: 72,500 23%; F: % 63220 22%) in both sexes according to newly estimated new cancer cases and deaths by sex in the United States in 2020 (1). Non-small cell lung carcinoma (NSCLC) accounts for 85% of lung cancer cases and is the most frequent primary site for brain metastasis (BM) (1–3). The relative incidence of BMs accounts for 40% of all NSCLC patients and is increasing with the development of advanced imaging technology, targeted agents, and immunotherapy (IT) (3–5). Radiation therapy (RT) has been predominantly used for the management of BMs (5–13).

BM patients with a high intracranial burden are primarily offered whole brain radiation therapy (WBRT). Generally, stereotactic radiosurgery (SRS) has been restricted to patients with up to 3 BMs. However, recent trends indicate that SRS/stereotactic radiotherapy (SRT) has been increasingly offered to patients with >3 BMs with WBRT used as salvage therapy (5, 9, 10, 12). In BM patients with NSCLC, a combination of the two may result in improved outcomes compared to being treated with either of the two (5, 7–12). Next-generation tyrosine kinase inhibitors (TKIs) and immune checkpoint blockades (ICBs) have shown efficacy in treating BMs (14, 15). The addition of up-front radiotherapy to TKIs may improve outcomes compared with TKI alone in EGFR-mutated NSCLC (14). Despite increased usage of SRS in recent times, BM patients are still managed with WBRT alone because of multiple brain lesions at presentation and the feasibility of SRS treatment (13).

ICB targeting the CTLA-4 and PD-1 checkpoint pathways has shown significant improvement in the survival of NSCLC patients (16). As a result, anti-PD-1/PD-L1 therapy has been approved as a first-line or second-line monotherapy treatment or given in combination with chemotherapy (16). Anti-PD-1 monoclonal antibodies (nivolumab/pembrolizumab) as monotherapy have also displayed an intracranial response with an objective response rate (ORR) of 9–30% in patients with NSCLC BMs (15, 17, 18). A rationale has been developed for the combination of immune checkpoint inhibitors (ICIs) and RT to seek synergistic anti-cancer responses (19). So far, there are limited reports of improvement in outcome with a combination of the two treatments (20–25). Hence, we are attempting to conduct a retrospective review involving NSCLC patients with BM to analyze the addition of anti-PD-1 therapy to WBRT compared to RT alone.

## Methods and Materials

### Patient Selection

A total of 70 stage IV non-small cell lung cancer patients with newly diagnosed brain metastases (≥ three BMs), and who had received WBRT alone (n=41) or in combination with anti-PD-1 therapy (n=29) for BMs during the time period between January 2016 and January 2021 at the “Shenzhen People’s Hospital, The First Affiliated Hospital Of Southern University Of Science And Technology, Shenzhen, China”, and “The First Affiliated Hospital Of Wenzhou Medical University, Wenzhou, China”, were identified by conducting a retrospective review of the database following ethics approval from the two hospitals. All the included patients had developed brain metastases after being previously treated with first line platinum-based chemotherapy at initial lung cancer diagnosis. Only six patients in control group and none in the anti-PD-1 group were diagnosed with synchronous brain metastases who were offered first line platinum-based chemotherapy or anti-EGFR therapy along with WBRT to the brain. The remaining patients in control group were offered second line docetaxel chemotherapy and/or anti-EGFR treatment for systemic disease, and WBRT for brain metastases. Patients in the anti-PD-1 group only received anti-PD-1 antibody treatment (nivolumab) that was started within 30 days of WBRT induction. At subsequent disease progression, all patients were offered best supportive care. WBRT was delivered with a median dose of 30 Gy/10 F. Clinicopathological information and follow-up time for all patients were recorded and are presented in **Table 1**. Written informed consent for participation was obtained from the patients or their guardians according to the Declaration of Helsinki (26). The STROBE guidelines for cohort studies were followed for reporting (27).

**Table 1:** Baseline characteristics of included patients.

Patient characteristics	Total	WBRT plus Anti-PD-1	WBRT alone	P value
No. of patients	70 (100%)	29 (41%)	41 (59%)	
**Age**	58.4 **±** 11.3	60 **±** 9.7	57 **±** 12.3	0.355
<60	35 (50%)	14 (40%)	21 (60%)	0.808
≥60	35 (50%)	15 (43%)	20 (57%)	
**Sex**				
Male	41 (59%)	20 (49%)	21 (51%)	0.138
Female	29 (41%)	9 (31%)	20 (69%)	
**Smoking**				
Never	50 (71%)	19 (38%)	31 (62%)	0.357
Former/current	20 (29%)	10 (50%)	10 (50%)	
**NSCLC pathology**				
Adenocarcinoma	60 (86%)	22 (37%)	38 (63%)	0.114
Squamous/Large cell	8 (14%)	6 (75%)	2 (25%)	
**Pathology differentiation**				
Well	28 (40%)	10 (36%)	18 (64%)	0.428
poor	42 (60%)	19 (45%)	23 (55%)	
**KPS**				
≤80	39 (56%)	16 (41%)	23 (59%)	0.939
90-100	31 (44%)	13 (42%)	18 (58%)	
**BMI** (Median (min, max)	22 (16, 31)	23 (16, 29)	22 (18, 31)	
Normal (<25)	59 (84%)	24 (41%)	35 (59%)	0.768
Overweight (≥25)	11 (16%)	5 (45%)	6 (55%)	
**Genetic status**				
EGFR +	30 (43%)	5 (17%)	25 (83%)	0.004*
EGFR -	31 (44%)	16 (52%)	15 (48%)	
Unknown	9 (13%)	8 (89%)	1 (11%)	
**No. of Metastatic organs**				
Brain only	28 (44%)	14 (50%)	14 (50%)	0.235
Extracranial metastasesΨ	42 (56%)	15 (36%)	27 (64%)	
**Follow-up**	16.9 **±** 8.0	18.8 **±** 6.9	15.5 **±** 8.5	0.090
Median (min, max)	15 (2, 36)	18 (9, 36)	14 (2, 35)	

WBRT, whole-brain radiotherapy; PD-1, programmed cell death protein-1; NSCLC, non-small cell lung carcinoma; KPS, Karnofsky performance status; BMI, body mass index; HR, hazard ratio; CI, confidence interval.

Data are presented as mean ± SD or frequency (%), unless indicated otherwise.

*Statistical significance (p<0.05).

ΨExtracranial metastatic organs included liver, bone, and breast metastases.

### Follow Up and Endpoints

Follow-up included clinical evaluation and radiological imaging tests (CT and MRI) obtained at 3-, 6-month-, and 1-year intervals. Overall survival (OS) was termed as the primary endpoint and defined as the time from BM diagnosis to death. Progression-free survival (PFS) was termed as the secondary endpoint and defined as the time from BM diagnosis to disease progression on clinical and radiological evaluation during follow-up or death following treatment induction. Progressive disease was defined according to the RECIST 1.1, in which new BM occurrence was also characterized as disease progression (28). Adverse events experienced by patients after receiving treatment were also assessed and graded according to the Common Terminology Criteria for Adverse Events (CTCAE) version 5.0 criteria (29).

### Statistical Analysis

Median survival time and confidence intervals were obtained for OS and PFS using Kaplan-Meier analysis available in SPSS software version 26. The log-rank test was used to determine statistical differences between the groups’ OS and PFS outcomes. To determine the differences between the cohorts, Chi-square test/Fisher exact test for categorical covariates, and t-tests for numerical covariates were applied if tests of normality and homogeneity of variance were satisfied. Otherwise, the Mann-Whitney U test or Kruskal-Wallis H test was used. The Cox proportional hazard model was used for univariate and multivariate factor analyses. Factors with p-values less than 030 (p<0.30) were selected for multivariate analysis.

## Results

### Patients’ Characteristics

Our retrospective review included 70 patients with stage IV NSCLC and ≥ three BMs. All patients were treated with WBRT between 2016 and 2021. Of the 70 NSCLC BM patients, 29 (41%) received a median number of 6 cycles of anti-PD-1 monoclonal antibody treatment (nivolumab) within 30 days of WBRT initiation. The entire cohort was followed up for an average of 17 months (standard deviation, **±** 8.0 m). The mean age of the entire cohort was 58.4 years (standard deviation, **±** 11.3 y). The majority of the patients in the WBRT alone group were EGFR+ (25 *vs*. 5), and the difference was significant between the treatment groups according to EGFR status (*p=0.004*). No significant association was found between the treatment groups for other baseline characteristics such as age, sex, smoking status, histopathology, tumor differentiation, Karnofsky performance status (KPS), number of metastatic organs, and follow-up duration (**Table 1**) BMI.

### Overall Survival

**Figure 1:**
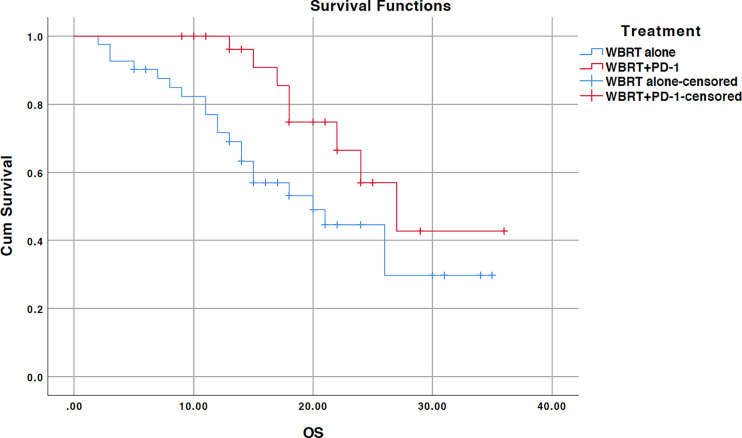
Kaplan-Meier overall survival (OS) curve for treatments; WBRT alone (No PD-1), and WBRT plus PD-1 inhibition therapy (WBRT+PD-1). Cum, cumulative.

The median survival for the entire cohort was 24 months (95% CI, 19.5–28.5) (**Figure 1**). The median survival time for the WBRT alone cohort was 20 months (95% CI, 11.6–28.3) and 27 months (95% CI, 19.5–28.5) for the WBRT plus anti-PD-1 cohort. OS was significant for the treatment difference (*p=0.035*). In the univariate analysis, age, sex, pathohistological type, pathological differentiation, KPS performance status, body mass index (BMI), and presence of extracranial metastatic sites had no impact on survival (**Table 2**). Additional anti-PD-1 antibody administration was the only predictor of OS identified on univariate analysis. Other factors that showed a close relationship with OS included sex (*p=0.095*) and histopathologic differentiation (*p=0.075*). These three factors were included in the multivariate analysis. Only the combined treatment remained significant for predicting OS on multivariate analysis.

**Table 2:** Univariate and multivariate analysis for overall survival.

Predictors	Comparators	Univariate		Multivariate	
		HR (95% CI)	Significance	HR (95% CI)	Significance
**Treatment group**	WBRT plus anti-PD-1	0.43 (0.19-0.97)	p=0.043*	0.41 (0.18-0.96)	p=0.040*
	WBRT alone				
**Age**	<60	0.78 (0.37-1.63)	*p=0.516*		
	≥60				
**Sex**	Female	0.53 (0.25-1.11)	***p=0.095***	0.68 (0.32-1.45)	*p=0.322*
	Male				
**Smoking**	Never	1.49 (0.67-3.30)	*p=0.322*		
	Former/Current				
**NSCLC pathology**	Adenocarcinoma	0.67 (0.31-1.45)	*p=0.314*		
	Squamous/Large cell				
**Path. differentiation**	Well	2.07 (0.92-4.64)	***p=0.075***	2.17 (0.96-4.95)	***p=0.063***
	poor				
**KPS**	≤80	1.14 (0.55-2.36)	*p=0.725*		
	90-100				
**BMI**	Normal (<25)	27.4 (0.45-1662)	*p=0.416*		
	Overweight (≥25)				
**Genetic status**	EGFR +	1.01 (0.45-2.26)	*p=0.979*		
	EGFR -				
**No. of Metastatic organs**	Brain only	0.88 (0.41-1.89)	*p=0.754*		
	Extracranial (1/2)**^Ψ^**				

WBRT, whole-brain radiotherapy; PD-1, programmed cell death protein-1; NSCLC, non-small cell lung carcinoma; KPS, Karnofsky performance status; BMI, body mass index; HR, hazard ratio; CI, confidence interval.

*Statistical significance (p<0.05).

**^Ψ^** Extracranial organs other than primary organ (lung) included liver, bone, and breast.

Bold font is used in univariate analysis for factors with p<0.30 and are selected for multivariate analysis. Bold font in multivariate analysis indicates close association.

**Figure 2:**
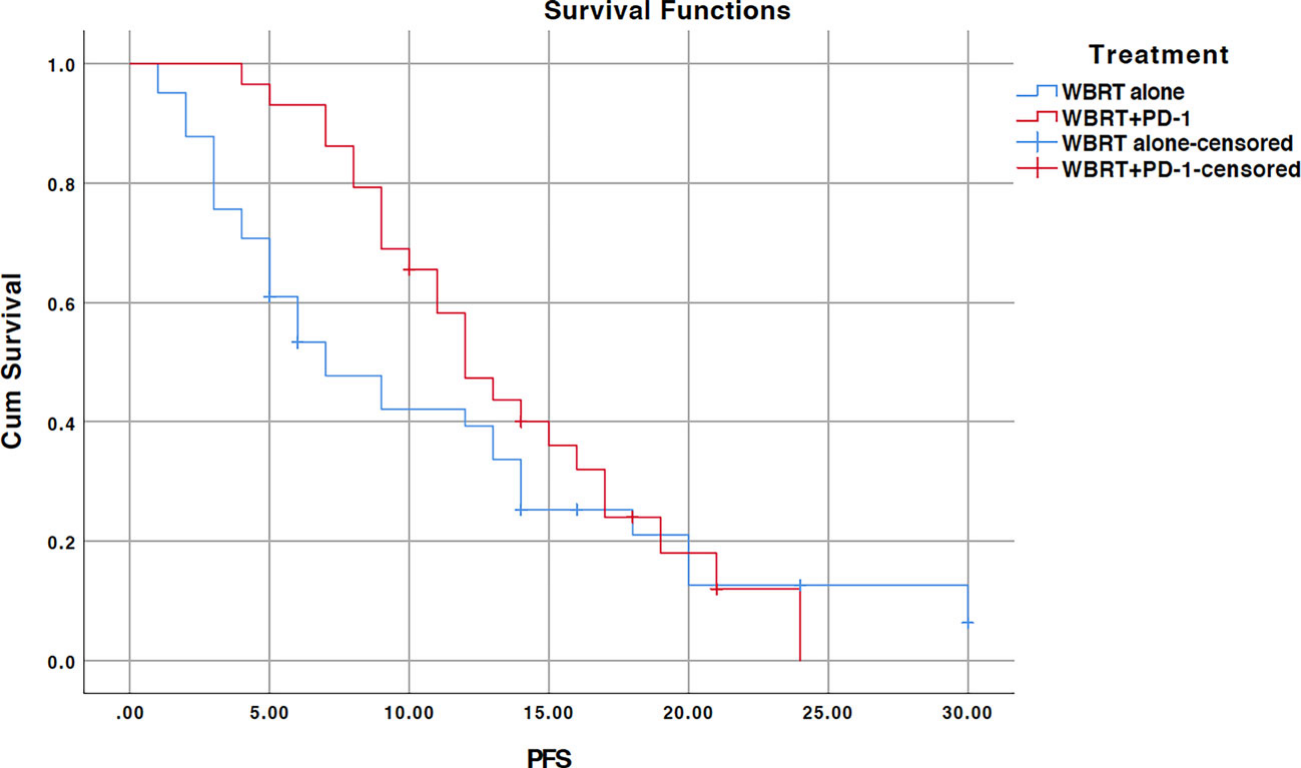
Kaplan-Meier overall survival (OS) curve for treatments in EGFR wildtype NSCLC subgroup; WBRT alone (No PD-1), and WBRT plus PD-1 inhibition therapy (WBRT+PD-1). Cum, cumulative.

We further performed subgroup analysis as there was a significant difference between two cohorts according to the EGFR mutation status (*p=0.004*). In subgroup analysis, significant improvement in OS for the treatment difference was unraveled for NSCLC patients negative for EGFR mutation (n=31, *p=0.012*). WBRT alone cohort (n=15) demonstrated a median OS of 15 months (95% CI, 11.5–18.4) while median OS for WBRT plus anti-PD-1 cohort (n=16) was not reached as shown in **Figure 2**. There was no difference when analysis was restricted to EGFR positive NSCLC patients (*p=0.096*). Median OS couldn’t be calculated as no events were reported in WBRT plus anti-PD-1 cohort (n=5) as illustrated in **Supplementary Figure 1**.

### Progression-Free Survival

**Figure 3:**
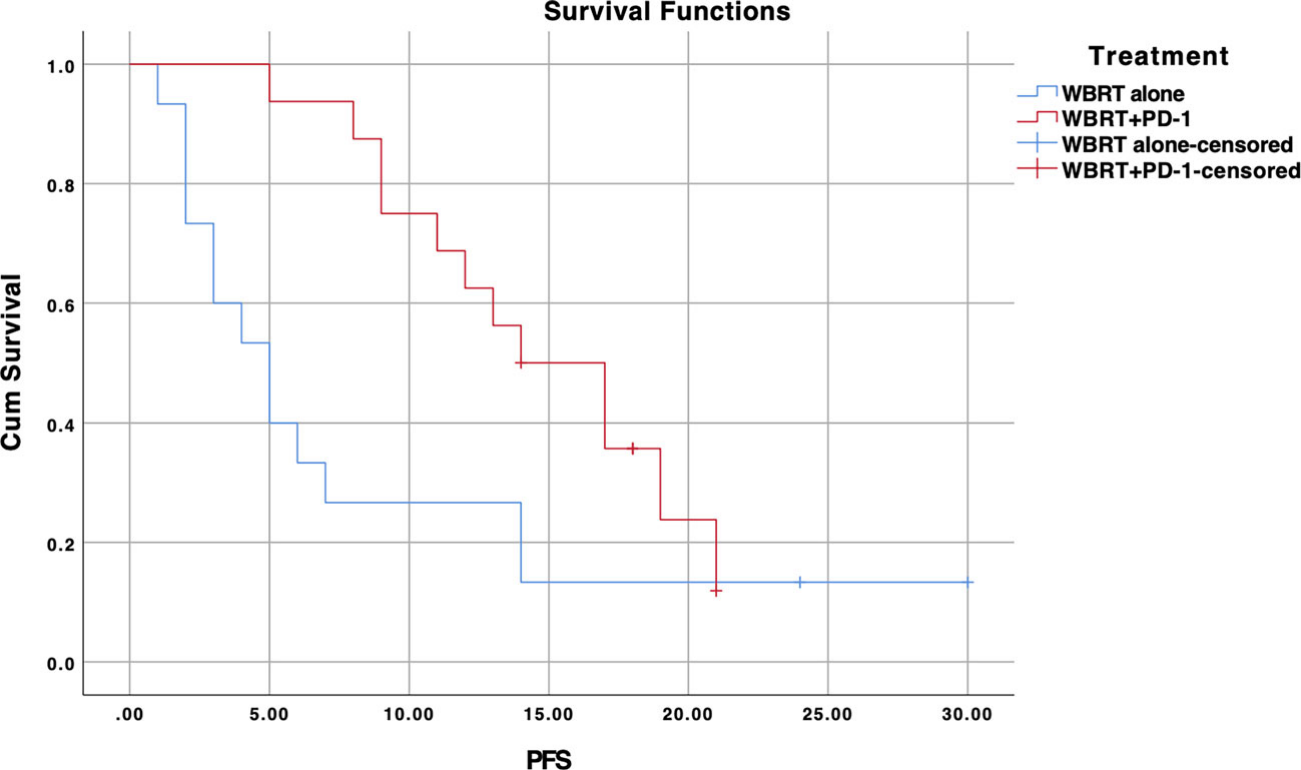
Kaplan-Meier progression-free survival (PFS) curve for treatments; WBRT alone (No PD-1), and WBRT plus PD-1 inhibition therapy (WBRT+PD-1). Cum, cumulative.

The median PFS for the entire cohort was 11 months (95% CI, 8.4–13.6) (**Figure 3**). The median PFS for the WBRT alone cohort was 7 months (95% CI, 3.7–10.3) and 12 months (95% CI, 9.4–14.5) for the WBRT plus anti-PD-1 cohort. The difference between the median PFS of the treatments was not significant (*p=0.247*). On univariate analysis, pathological differentiation (*p=0.021*) and KPS score (*p=0.047*) appeared to be predictive of better PFS. Both predictors lost statistical significance in the multivariate analysis (**Table 3**). Treatment (*p=0.061*), smoking (*p=0.066*), and pathological differentiation (*p=0.088*) showed close association with PFS on multivariate analysis.

**Table 3:** Univariate and multivariate analysis for progression-free survival.

Predictors	Comparators	Univariate analysis		Multivariate analysis	
		HR (95% CI)	Significance	HR (95% CI)	Significance
**Treatment group**	WBRT plus anti-PD-1	0.73 (0.43-1.25)	***p=0.264***	0.56 (0.30-1.02)	***p=0.061***
	WBRT alone				
**Age**	<60	0.87 (0.52-1.49)	*p=0.632*		
	≥60				
**Sex**	Female	0.66 (0.39-1.12)	***p=0.127***	0.63 (0.35-1.12)	*p=0.115*
	Male				
**Smoking**	Never	1.36 (0.75-2.47)	***p=0.301***	1.88 (0.95-3.69)	***p=0.066***
	Former/current				
**NSCLC pathology**	Adenocarcinoma	0.70 (0.37-1.33)	***p=0.274***	0.74 (0.37-1.48)	*p=0.398*
	Squamous/Large cell				
**Path. differentiation**	Well	1.91 (1.10-3.30)	***p=0.021***	1.69 (0.92-3.11)	***p=0.088***
	poor				
**KPS**	≤80	1.71 (1.00-2.89)	***p=0.047***	1.59 (0.90-2.82)	*p=0.107*
	90-100				
**BMI**	Normal (<25)	1.08 (0.77-1.63)	***p=0.658***		
	Overweight (≥25)				
**Genetic status**	EGFR +	0.96 (0.54-1.68)	*p=0.886*		
	EGFR -				
**No. of Metastatic organs**	Brain only	0.80 (0.46-1.38)	*p=0.428*		
	Extracranial (1/2)**^Ψ^**				

WBRT, whole-brain radiotherapy; PD-1, programmed cell death protein-1; NSCLC, non-small cell lung carcinoma; KPS, Karnofsky performance status; BMI, body mass index; HR, hazard ratio; CI, confidence interval.

Statistical significance (p<0.05).

**^Ψ^** Extracranial organs other than primary organ (lung) included liver, bone, and breast.

Bold font is used in univariate analysis for factors with p<0.30 and are selected for multivariate analysis. Bold font in multivariate analysis indicates close association.

**Figure 4:**
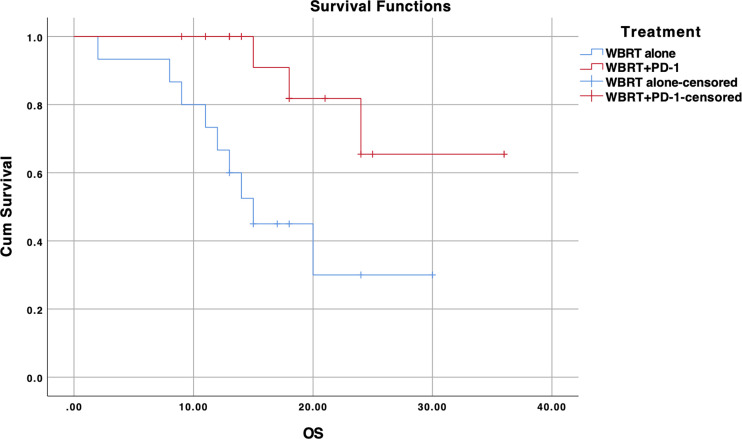
Kaplan-Meier progression-free survival (PFS) curve for treatments in EGFR wildtype NSCLC subgroup; WBRT alone (No PD-1), and WBRT plus PD-1 inhibition therapy (WBRT+PD-1). Cum, cumulative.

In subgroup analysis, NSCLC patients lacking EGFR mutation (n=31) showed significant improvement in PFS for the treatment difference (*p=0.037*) (**Figure 4**). WBRT plus anti-PD-1 cohort demonstrated a median PFS of 14 months (95% CI, 9.2–18.7) as compared to 5 months in WBRT alone (95% CI, 2.5–7.5). There was no difference when analysis was restricted to EGFR positive NSCLC patients (median PFS for WBRT alone (n=25): 9 months (95% CI, 3.6–14.4) *vs*. 12 months (95% CI, 8.0–15.9) for WBRT plus anti-PD-1 (n=5), *p=0.510*) (**Supplementary Figure 2**).

### Adverse Events

Overall, 47 patients (67%) experienced at least one adverse event. In the WBRT plus anti-PD-1 group, 18 (62%) experienced 34 (47.8%) adverse events, while 27 (65.8%) patients in the WBRT alone cohort suffered from 37 (52%) adverse events. There was no significant difference between the treatment cohorts in terms of adverse events (ORR, 0.94 [95% CI, 0.44–2.02), *p=0.879*). Most adverse events were of grade 1 or 2 severity. Only two grade 3 events were reported, and both were in patients receiving additional anti-PD-1 therapy. Rash, pruritis, hyperthyroidism, and hypothyroidism were mainly observed in the combined treatment group. The reported adverse events are listed in **Table 4**.

**Table 4:** Adverse events experienced by two cohorts.

Factors	Total			WBRT plus Anti-PD-1			WBRT alone		
Participants	47			18			27		
Odds ratio	0.94 (95% CI, 0.44-2.02), *p=0.879*								
Adverse events	71			34			37		
Odds ratio	1.29 (95% CI, 0.66-2.52). *p=0.441*								
***Adverse events and relative grading frequency***									
**AEs\Grades**	**Total**			**WBRT plus Anti-PD-1**			**WBRT alone**		
	**All Grades**	**G2**	**G3**	**All Grades**	**G2**	**G3**	**All Grades**	**G2**	**G3**
Fatigue	12	6	0	5	2	0	7	4	0
Nausea/vomiting	12	3	1	5	0	1	7	3	0
Low appetite	11	6	0	4	2	0	7	4	0
Headaches	11	6	0	3	1	0	8	5	0
Hypothyroidism*	8	1	0	6	1	0	2	0	0
Cerebral edema	5	2	0	4	1	0	1	1	0
Liver toxicity	2	0	0	1	0	0	1	0	0
Rash/pruritis*	2	2	0	2	2	0	0	0	0
Hyperthyroidism*	2	0	0	2	0	0	0	0	0
Gait problems	1	0	0	0	0	0	1	0	0
Mental status	2	1	0	1	1	0	1	0	0
Hyponatremia	2	0	1	1	0	1	1	0	0
Radiation Necrosis	1	0	0	0	0	0	1	0	0
Total	71	27	2	34	10	2	37	19	0

WBRT, whole-brain radiotherapy; PD-1, programmed cell death protein-1; G, grade; CI, confidence interval.

*Predominantly observed in patients receiving anti-PD-1 therapy.

## Discussion

Lung cancer is often diagnosed at an advanced stage, with a 5-year survival rate of 5% (1). Advanced stage NSCLC patients lacking molecular markers are offered chemotherapy alone or chemotherapy in combination with immune checkpoint inhibitors (ICIs) as first-line treatment (16). Moreover, the addition of stereotactic ablative radiotherapy (SABR) to IT has also been tested in metastatic NSCLC (30, 31). This combination has demonstrated abscopal responses in metastatic sites and delayed disease progression (30, 31). In fact, multiple-lesion radiotherapy has shown to enhance the efficacy of PD-1 checkpoint inhibitors as compared to single-lesion receiving radiotherapy indicating synergism (32). However, the efficacy of this combination of RT and ICI in the brain remains to be elucidated. Our retrospective analysis of 70 NSCLC patients with multiple BMs (>3) revealed a better PFS (though not significant) and significantly improved OS in patients receiving concurrent treatment without any increase in toxicity.

The benefit in response can be explained based on preclinical and clinical evidence. Clinical response to ICIs is predicated on PD-L1 expression and the density of tumor-infiltrating lymphocytes (TILs), with a correlation between the primary tumor and BMs in lung adenocarcinoma (33–37). Moreover, RT promotes PD-L1 expression in metastatic sites by improving antigen presentation and tumor-specific immunity, which could be further augmented by ICIs to overcome the acquired resistance to RT (38–40). Third, local damage caused to the blood-brain-barrier during RT could also provide a window for IT drugs to be effective in the brain (41). For example, secondary analysis of the KEYNOTE-001 phase I trial demonstrated a comparatively superior PFS (median PFS: 4.4 *vs*. 2.1 months) and OS (median OS: 10.7 *vs*. 5.3 month) in the cohort that had previously received RT to the brain or extracerebral in addition to pembrolizumab, as compared to pembrolizumab alone (21). Moreover, the two concurrently administered treatments showed superior efficacy compared to use of only one of either treatment. In a retrospective study of BM patients (n=260) with primary tumor sites such as NSCLC, melanoma, and renal cell carcinoma (RCC), the use of ICIs with SRS/SRT (n=79) improved the median OS compared to SRS/SRT alone (14.5 *vs*. 12 months) (22). The improvement in OS (24.7 months) was significantly higher for the concurrent (within 2 weeks) IT (CI) cohort (n=28) than in the non-concurrent IT (nCI) cohort (HR 2.40, *p=0.006*) and SRS/SRT alone group (HR 2.69, *p=0.002*) (22). The median survival for concurrent group (n=28, median OS= 24.7 months) reported in the study by Chen et al. was similar to our study (n=29, median OS=27 months) (22). Likewise, no statistical difference in PFS (PFS: CI=2.3 *vs*. nCI=2.3 *vs*. SRS alone=3.7 months) was revealed between the cohorts (7 *vs*. 12 months, *p=0.264*) (22). However, the melanoma patients were prevalent in concurrent group (83%) in their study, which was also significant predictor of survival on multivariate analysis (HR 2.7, 95% CI: 1.6–4.7 for NSCLC; 3.6, 95% CI: 1.4–8.3 for RCC) (22). Melanoma BM patients (n=48, median OS=394 days) were also a significant driver of OS from the first anti-PD-1 therapy in comparison to NSCLC (n=79, median OS=192 days) and RCC (n=10, median OS=121 days) in a retrospective study by Pike et al. (23). In their study, among 59 BM patients who received RT following PD-1 inhibition, 25 continued to receive anti-PD-1 therapy for a median of 179 days and showed an improved median survival (additional 238 days) (23). In another retrospective matched cohort study of NSCLC-derived BM, the concurrent use of ICI (n=17, BMs=45) within 3-months of SRS provided a significantly rapid regression of BM (2.5 *vs*. 3.1 months, *p<0.0001*) and improved CNS complete response (CR) [8/16 (50%) *vs*. 5/32 (15.6%), *p=0.012*] compared to SRS alone (n=34, BM =92) (24). However, this benefit was not translated clinically in terms of PFS (HR 2.18; 95% CI, 0.72–6.62; *p=0.11*) and OS (HR 0.99, 95% CI: 0.39–2.52, *p=0.99*). Similarly, no statistical difference in median survival was found between the IT group (n=39) and CT group (n=46) in a retrospective study of 85 NSCLC patients with BMs (median OS: 10 *vs*. 11.6 months, *p=0.23*) (25). However, lesion shrinkage was significantly higher in the IT group than in the CT group in a subset of patients with lesion volume > 500 mm^3^ (90% *vs*. 47.8%, *p=0.001*). In both of these studies, even though no survival advantage was achieved, intracranial responses were observed with the combined approach. Similarly, the 6-month distant brain control rate for the before/concurrent cohort was significant compared to the post-RT cohort (57% *vs*. 0%, *p=0.05*) in a small cohort of NSCLC BM patients (n=17) receiving anti-PD-1 (nivolumab/durvalumab) and/or SRS/fractionated stereotactic radiation therapy (42). The timing (before/concurrent *vs*. after) was also significant for OS on univariate analysis (HR 9.2, 95% CI: 1.9–65.3, *p=0.006*) but not on multivariate analysis (HR 3.6; 95% CI, 0.74–26.9; *p=0.11*) (42). Another retrospective study on metastatic NSCLC patients revealed that delivering radiation before (6 months) or during/after (3 months) nivolumab administration was not associated with better OS or PFS (43). These outcomes endorse the observation that a window of at least 14 days was essential for palliative RT prior to the administration of nivolumab to take advantage of the RT-induced tumor antigenic stimulation effect (44, 45). Preclinical evidence also suggests that concurrent RT/anti-PD-1 inhibition may induce better anti-cancer effects compared to RT undertaken prior to PD-1 inhibition (30). Therefore, the literature implies that these patients may benefit from a combination of both treatments.

Investigation of factors affecting OS or PFS revealed no impact for several factors including age, gender, smoking, pathology, KPS, and the presence of EGFR mutation and extracranial metastases. Nonetheless, histopathologic differentiation showed slight association with worst PFS and OS. Likewise, smoking and KPS have also shown to negatively impact PFS on univariate and/or multivariate analysis. Importantly, WBRT alone group had more EGFR-positive participants, which may have confounded survival advantage as EGFR inhibitor plus WBRT treatment yields better survival compared to WBRT alone in NSCLC BM patients (14). For this reason, subgroup analysis was performed which revealed a significant improvement in PFS and OS for EGFR negative NSCLC patients. This result in concordance to a previously published meta-analysis comprising seven randomized controlled trials, in which immune checkpoint blockade had resulted in significantly better PFS and OS as compared to chemotherapy in EGFR wild-type stage IV NSCLC patients (PFS: HR 0.83, 95% CI 0.73-0.95); OS: HR 0.67, 95% CI 0.60-0.76; p<00001) (46). In this meta-analysis, EGFR mutant responded better to chemotherapy as opposed to immunotherapy in terms of PFS and OS. Likewise, no significant improvement in PFS and OS was found in EGFR positive patients in our study. Nonetheless, the cohort for combined treatment (n=5) was very small and further investigation would be required to establish presence or lack of benefit for the combined treatment in EGFR positive NSCLC patients with brain metastases.

Our study showed the safety of the combined approach with no increase in toxicity. Other studies have also revealed that the combination of ICIs and RT does not lead to an increase in toxicity compared to RT alone or ICI alone (47). In a single-center secondary analysis of a phase 1/2 trial (n=10), a combination of palliative RT (3DCRT, 79% and SRS–SRT, 21%; 28 Gy/5 fraction) plus durvalumab (10 mg/kg every 2 weeks *via* intravenous infusion) led to no grade 3 RT-related adverse events (AEs) (NCT01693562) (47). All AEs were transient and manageable according to the standard guidelines (47). Concurrent ICI (nivolumab within 3-month of RT) was also not associated with any increase in the rate of radiation necrosis or intratumoral hemorrhage in NSCLC-derived BM patients (5.9% *vs*. 2.9% in ICI-naive cohort, *p=0.99*) (24). There were no significant differences in the rates of all-grade AEs and grade ≥3 AEs between the ICI-naive (n=113) and ICI-treated NSCLC BM patients (n=50) across different cranial RT types (grade ≥3 AEs in 8% *vs*. 9% for SRS, *p=1.00*; 8% *vs*. 10% for WBRT, *p=0.71*) (48). Additionally, there was no difference in AE rates based on the timing of ICI administration with respect to RT.

Our study is limited by inherent biases of retrospective research design, which include selection, information, recall, and/or observation biases (49). The small sample size also limits our study. There is also chronological bias since WBRT alone participants were diagnosed earlier, whereas the PD-1 inhibition therapy group participants were diagnosed later. Moreover, certain outcomes were not reported such as intracranial response rate and subsequent therapies undertaken after completion or withdrawal from either treatment.

## Conclusions

Our results indicate that concurrent WBRT and anti-PD-1 therapy may enhance OS in NSCLC patients with BM particularly in EGFR negative patients. The addition of anti-PD-1 therapy to RT may not increase the toxicity. Further studies are warranted to validate and elucidate the effect of using the treatments in combination.

## Data Availability Statement

The original contributions presented in the study are included in the article/**Supplementary Material**. Further inquiries can be directed to the corresponding authors.

## Ethics Statement

Ethics approval was obtained from the Ethical review board of “Shenzhen People’s Hospital, The First Affiliated Hospital Of Southern University Of Science And Technology, Shenzhen, China”, and “The First Affiliated Hospital Of Wenzhou Medical University, Wenzhou, China. The patients/participants provided their written informed consent to participate in this study.

## Author Contributions

GXL and GL provided the data. GXL and MK wrote the manuscript. All authors approved the design, data collection, data analysis, and final manuscript for publication.

## Funding

The Natural Science Foundation of Shenzhen (No.JCYJ2017 0307095828424); Shenzhen Health and Family Planning System Research Project (No.SZBC2017024) were providing support for this work.

## Conflict of Interest

The authors declare that the research was conducted in the absence of any commercial or financial relationships that could be construed as a potential conflict of interest.

## Publisher’s Note

All claims expressed in this article are solely those of the authors and do not necessarily represent those of their affiliated organizations, or those of the publisher, the editors and the reviewers. Any product that may be evaluated in this article, or claim that may be made by its manufacturer, is not guaranteed or endorsed by the publisher.

## Supplementary Material

Supplementary Figure 1 Kaplan-Meier overall survival (OS) curve for treatments in EGFR mutant NSCLC subgroup; WBRT alone (No PD-1), and WBRT plus PD-1 inhibition therapy (WBRT+PD-1). Cum, cumulative.

Supplementary Figure 2 Kaplan-Meier progression-free survival (PFS) curve for treatments in EGFR mutant NSCLC subgroup; WBRT alone (No PD-1), and WBRT plus PD-1 inhibition therapy (WBRT+PD-1). Cum, cumulative.
