# Supplementary figures and images for: Radiation Plus Anti-PD-1 Therapy for NSCLC Brain Metastases: A Retrospective Study
Source: Front Oncol. 2021 Oct 21;11:742971. doi: 10.3389/fonc.2021.742971 (PMC8567143; doi:10.3389/fonc.2021.742971)

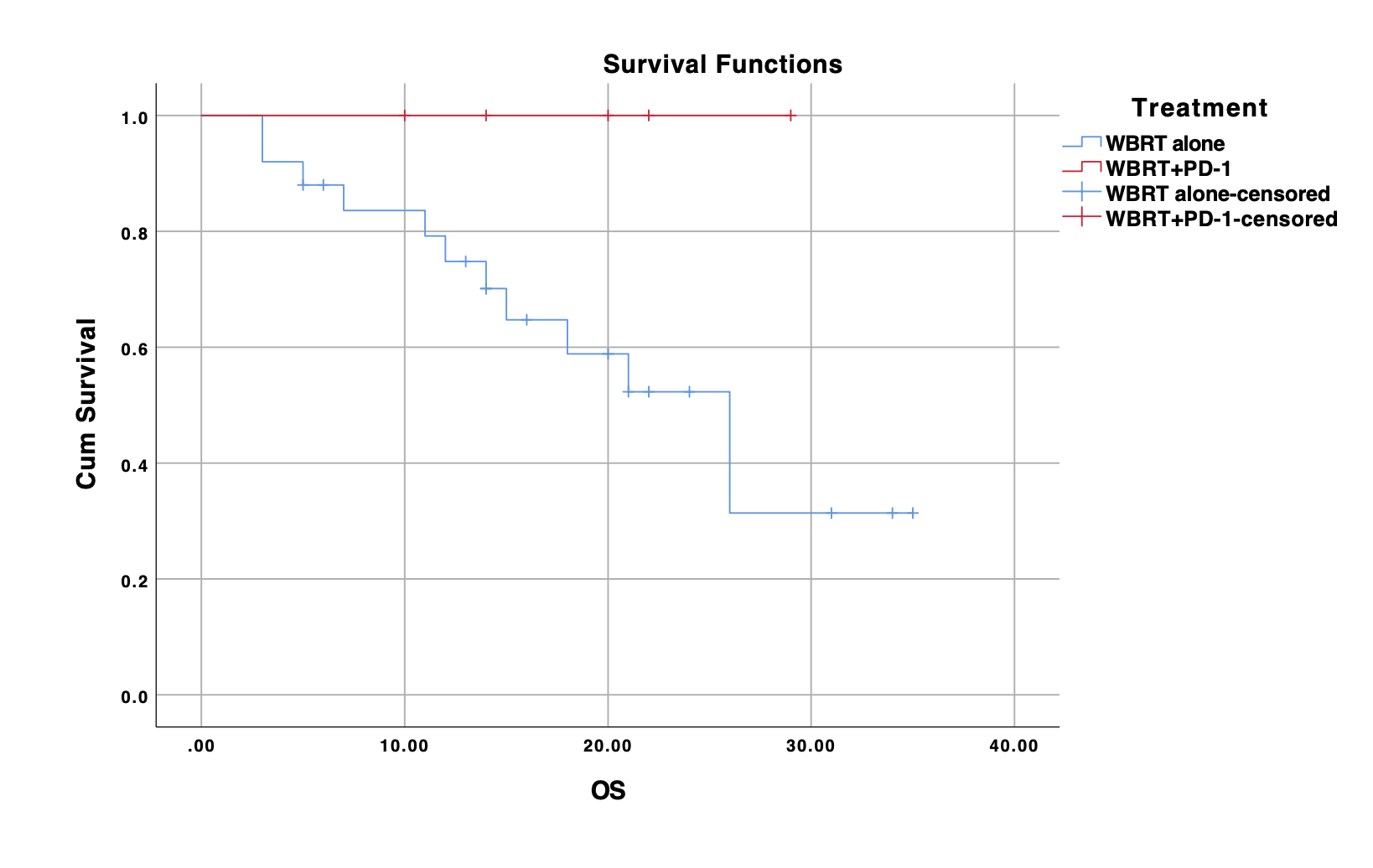

Supplement: Supplementary Figure 1 — Kaplan-Meier overall survival (OS) curve for treatments in EGFR mutant NSCLC subgroup; WBRT alone (No PD-1), and WBRT plus PD-1 inhibition therapy (WBRT+PD-1). Cum, cumulative. [file Image_1.jpeg]

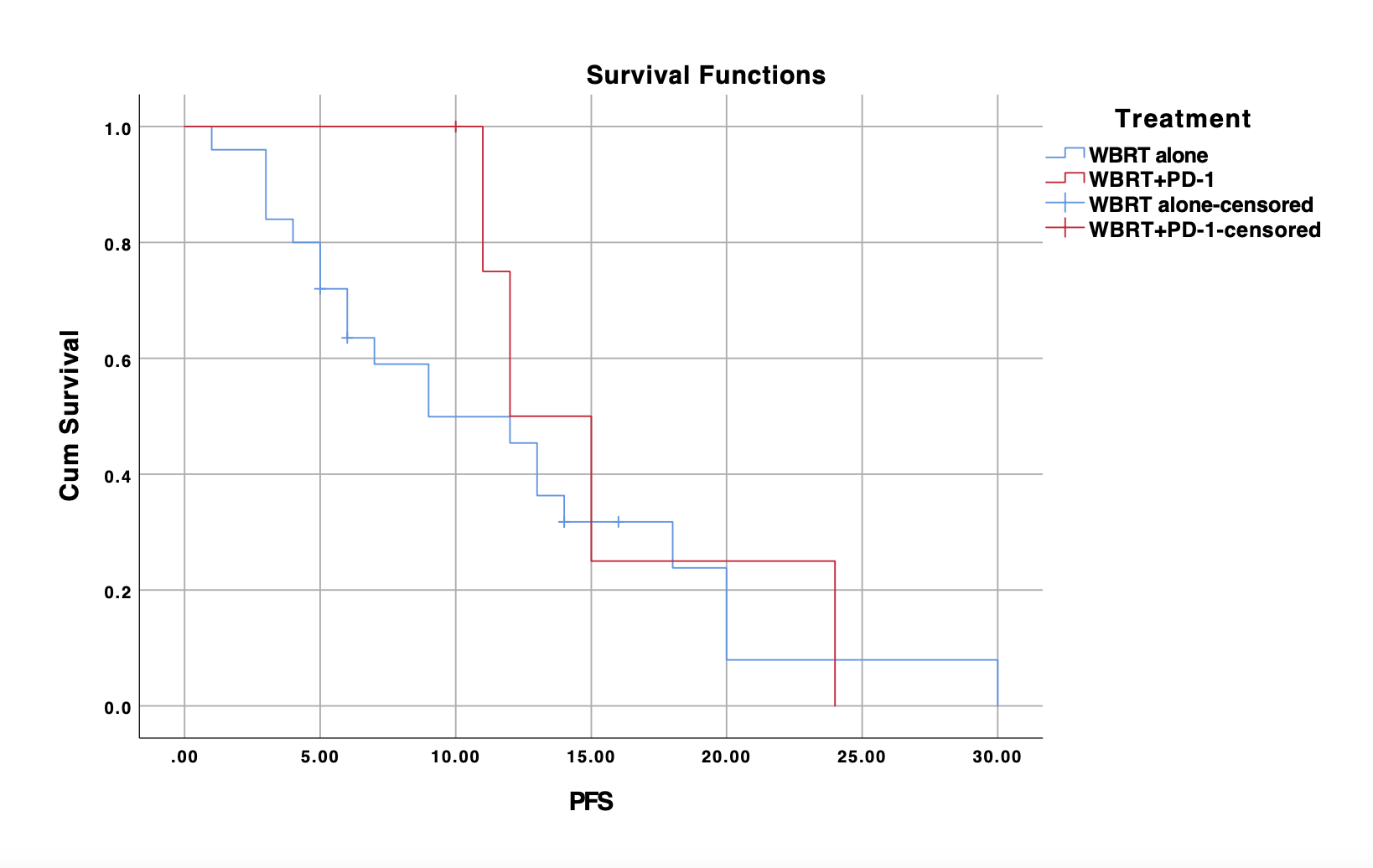

Supplement: Supplementary Figure 2 — Kaplan-Meier progression-free survival (PFS) curve for treatments in EGFR mutant NSCLC subgroup; WBRT alone (No PD-1), and WBRT plus PD-1 inhibition therapy (WBRT+PD-1). Cum, cumulative. [file Image_2.jpeg]
